# Ototoxic effects of antineoplastic drugs: a systematic review

**DOI:** 10.1016/j.bjorl.2021.02.008

**Published:** 2021-03-13

**Authors:** Fernanda Soares Aurélio Patatt, Laura Faustino Gonçalves, Karina Mary de Paiva, Patrícia Haas

**Affiliations:** a Universidade Federal de Santa Maria (UFSM), Santa Maria, RS, Brazil; b Universidade Federal de Santa Catarina (UFSC), Florianópolis, SC, Brazil

**Keywords:** Antineoplastic drugs, Hearing, Ototoxicity, Cisplatin, Exposure

## Abstract

**Introduction:**

Platinum-based chemotherapeutics play an important role in the treatment of cancer at different levels and are the most cited ototoxic agents when scientific evidence is analyzed.

**Objective:**

To present scientific evidence based on a systematic literature review, PRISMA, in order to systematize information on the ototoxic effects of using antineoplastic drugs.

**Methods:**

For the selection of studies, the combination based on the Medical Subject Heading Terms (MeSH) was used. The Medline (Pubmed), LILACS, SciELO, SCOPUS, WEB OF SCIENCE and BIREME databases were used, without restriction of language, period, and location. Evaluation of the quality of the articles was carried out, which included articles with a minimum score of 6 in the modified scale of the literature. The designs of the selected studies were descriptive, cohort, and cross-sectional, which were related to the research objective.

**Results:**

Three articles were included in this systematic review. The ototoxicity caused by cisplatin alone varied from 45% to 83.3%, while that caused by the use associated with carboplatin varied from 16.6% to 75%. There was a significant variation in the cumulative doses of these antineoplastic agents, both in isolated and in combination. Auditory changes, especially at high frequencies, were evident after completion of treatment.

**Conclusion:**

Auditory changes after the use of platinum-based antineoplastic drugs were found, however, there was an important heterogeneity regarding the frequency of ototoxicity and the cumulative dose of the drugs used.

## Introduction

Cancer is a public health problem and estimates indicate that 625 thousand new cases of cancer will occur in Brazil for each year of the 2020–2022 triennium.^1^ Worldwide, according to a projection by the International Cancer Research Agency (ICRA), affiliated to the World Health Organization (WHO), the incidence of cancer is expected to increase by up to 63% in the next 20 years, affecting more than 29 million new people in 2040.^2^

Antineoplastic chemotherapy is one of the systemic treatments for cancer that aims to treat malignant neoplasms and consists of the use of chemical substances alone or in combination, standing out as the preferential treatment for both the hematopoietic system and solid tumors, which may show regional or distant metastases. Antineoplastic or chemotherapeutic drugs interfere with the mechanisms of cell survival, proliferation, and migration, however, they act in a non-specific way, being able to harm both malignant and benign cells.^3^ Chemotherapy, as well as other cancer treatment modalities, may generate side effects for patients, and ototoxicity stands out.^4^, ^5^, ^6^, ^7^, ^8^

Ototoxicity is defined as a transient or permanent disorder of auditory and/or vestibular function induced by therapeutic substances,^9^ manifesting with hearing loss, tinnitus and/or vertigo. In cancer patients, it is associated with a subset of antineoplastic therapies that include chemotherapy with platinum, radiation or surgery involving the ear and vestibulocochlear nerve, in addition to supportive care agents, such as aminoglycoside antibiotics and loop diuretics, which may also contribute to ototoxicity.^10^

Platinum-based chemotherapy drugs play an important role in the treatment of cancer at different levels and are the most cited ototoxic agents when analyzing scientific evidence, with the outer hair cells of the cochlea being the most affected structures,^11^ leading to hearing loss and impairing social communication.^5^ The list of ototoxic antineoplastic drugs includes vinscristine, doxorubicin, gemcitabine, cyclophosphamide, farmorubicin, oxaliplatin,^12^, ^13^ carboplatin,^14^, ^15^ and cisplatin,^15^, ^16^, ^17^, ^18^, ^19^, ^20^, ^21^, ^22^, ^23^ with these drugs being generally used in combination of two to four drugs.

Among these drugs, cisplatin is the most used, and the effects on the cochlea may include sensorineural, bilateral, symmetrical, and irreversible hearing loss.^18^, ^19^, ^23^ The incidence of hearing loss in individuals treated with chemotherapy has an important variation in the literature due to factors such as frequency assessment, patient age, medication dosage, drug administration plan, and criteria adopted for hearing loss assessment.^23^ Other factors that can directly influence ototoxicity due to chemotherapy consist of tissue susceptibility to the drug, drug accumulation in the organ, inhibition of normal physiological functions, direct toxic effects on sensory terminal organs, ototoxic synergism, use of other concomitant ototoxic drugs and radiation treatment.^24^ The hearing impairment resulting from antineoplastic treatments by means of ototoxic drugs affects patients of different ages and, therefore, it is important that these patients undergo monitoring with audiological tests before, during, and after treatment.^11^, ^16^, ^17^

Based on the above, the present study aimed to present scientific evidence, based on a systematic review of the literature (PRISMA), on the ototoxic effects of antineoplastic drugs, aiming to answer the following guiding research questions: what are the effects and dose required for the ototoxicity of using antineoplastic drugs?

## Methods

### Protocol and registration

This systematic review was carried out according to the recommendations of the Preferred Reporting Items for Systematic reviews and Meta-Analyzes (PRISMA).^25^

The searches for scientific articles were carried out by two independent researchers in the electronic databases MEDLINE (Pubmed), LILACS, SciELO, SCOPUS, WEB OF SCIENCE, SCOPUS, WEB OF SCIENCE, and BIREME, without restriction of language, period, and location. In addition, a manual search was performed on the references of the articles included in the search and a search for gray literature on Google Scholar.

The research was structured and organized using the Population, Intervention, Comparison, Outcome and Study (PICOS) strategy, which represents an acronym for **P**opulation of interest or health problem (patients), **I**ntervention concerns antineoplastic agents, **C**omparison corresponds to drugs, **O**utcome refers to ototoxicity, and **S**tudy refers to types of studies included, which were: descriptive study, cross-sectional study, observational study, case reports, case-control studies, controlled clinical trials, and cohort studies (Table 1).

**Table 1:** Description of the PICOS components.

Acronym	Definition
P	Patients
I	Antineoplastic
C	Medication
O	Ototoxicity
S	Descriptive study
	Cross-sectional study
	Case reports
	Case-control studies
	Controlled clinical trials
	Cohort studies

Source: Developed by the authors.

### Research strategy

Descriptors were selected from a Health Sciences Descriptors (Descritores em Ciências da Saúde — DeCS) and Medical Subject Heading Terms (MeSH), considering the wide use of these tools by the scientific community to index articles in the PubMed database. In the search for descriptors, the adequacy for the other bases used was carried out.

At first, the following Boolean operators and combination of terms were proposed for the searches: (ototoxicity) AND (antineoplasic) AND (adults) AND (Childhood) AND (adolescent) AND (randomized controlled trial[pt] OR controlled clinical trial[pt] OR randomized controlled trials[mh] OR random allocation[mh] OR double-blind method[mh] OR singleblind method[mh] OR clinical trial[pt] OR clinical trials[mh] OR (“clinical trial” [tw]) OR ((singl*[tw] OR doubl*[tw] OR trebl*[tw] OR tripl*[tw]) AND (mask*[tw] OR blind*[tw])) OR ("latin square"[tw]) OR placebos[mh] OR placebo*[tw] OR random*[tw] OR research design[mh: noexp] OR follow-up studies[mh] OR prospective studies[mh] OR cross-over studies[mh] OR control*[tw] OR prospectiv*[tw] OR volunteer*[tw]) NOT (animal[mh] NOT human[mh]). The search was centered in October 2020.

### Eligibility criteria

The designs of the studies with potential for selection were descriptive, cross-sectional, observational, case-control, cohort, case reports, and controlled clinical trials. Studies were included without restriction of language, period, and location. Table 2 represents the inclusion and exclusion criteria developed in this research. The studies scored higher than 6 in the modified protocol by Pithon et al.^26^ to assess their quality.

**Table 2:** Summary of inclusion/exclusion criteria.

Inclusion criteria	
Design	Cross-sectional study
	Case-control studies
	Cohort studies
	Case reports
	Intervention studies
	Controlled clinical trials
Localization	Without restriction
Language	Without restriction

Exclusion criteria	
Design	Letter to the editor
	Guidelines
	Literature review
	Systematic review
	Narrative review
	Meta-analysis
Studies	Unclear studies
	Poorly described or inadequate
Form of publication	Abstract only

Source: Developed by the authors.

### Risk of bias

The quality of the methods used in the included studies was independently assessed by the reviewers (PH and LFG), according to the PRISMA recommendation.^25^ The assessment prioritized the clear description of the information. At this point, the review was carried out blindly, masking the names of the authors and journals, avoiding any potential bias and conflict of interest.

### Exclusion criteria

Studies published in the form of Letters to the Editor, guidelines, literature reviews, narrative reviews, systematic reviews, meta-analyzes, and abstracts were excluded. Studies that did not describe the specificities chosen by the researchers as an objective for this research or that were unclear were also excluded (Table 2).

### Data analysis

The extraction of data for the studies’ eligibility process was performed using a specific form for systematic review prepared by two researchers in Excel® Program, in which the extracted data were initially added by one of the researchers and then checked by the other researcher. Initially, they were selected according to the title; then, abstracts were analyzed and only those that were potentially eligible were selected. Based on the abstracts, the articles were selected for full reading and those that met all predetermined criteria were included. In case of disagreement between evaluators, a third evaluator made the decision on the eligibility of the study in question.

### Study selection

Initially, the eligibility reviewers (PH and LFG) were calibrated to perform the systematic review by FSAP and KMP. After calibration and clarification of doubts, the titles and abstracts were examined by two eligibility reviewers (PH and LFG), independently, who were not blind to the names of the authors and the journals. Those who presented a title within the scope, but the abstracts were not available, were also retrieved and analyzed in full. Subsequently, the eligible studies had the full text retrieved and evaluated. In the absence of an agreement between the reviewers, a third party (FSAP) was involved for the final decision.

### Collected data

After screening, the texts of the selected articles were reviewed and extracted in a standardized manner by two authors (PH and LFG) under the supervision of KMP and FSAP, identifying the year of publication, place of research, language of publication, type of study, objective, sample, method, result and conclusion of the study, frequency of ototoxicity, cumulative doses, age at diagnosis, mean time between the last dose of cisplatin and the hearing assessment, time of audiological monitoring.

### Clinical outcome

The clinical outcome of interest was to systematize information about the ototoxic effects of using antineoplastic drugs and observe the effective dose for such effects. Those who did not use this approach were not part of the literature review sample.

## Results

**Figure 1:**
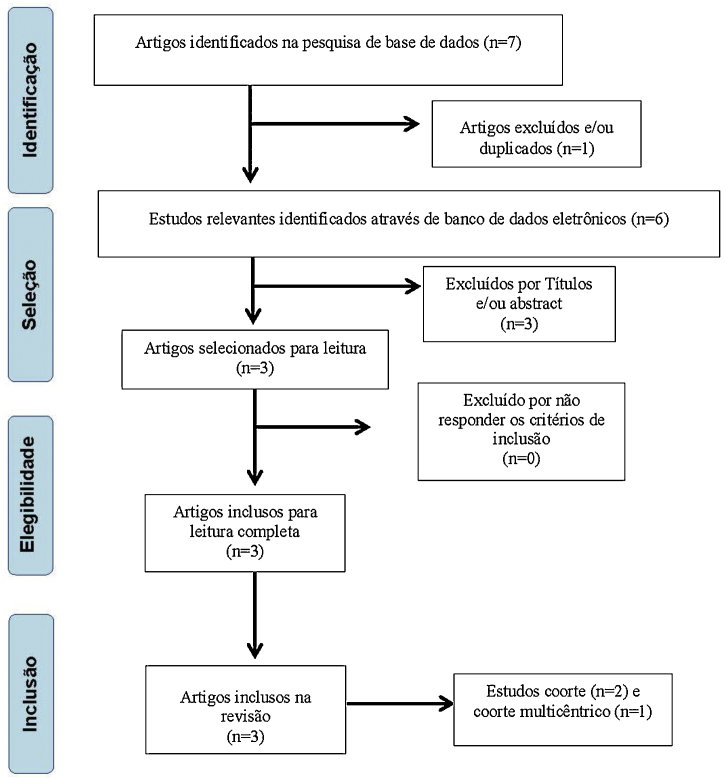
Flowchart of the articles search and analysis process.

Initially, 7 articles with potential for inclusion in this study were selected, with 6 remaining after exclusion by repetition. The titles were analyzed and 2 papers were excluded for not meeting the inclusion criteria proposed by these authors, leaving 4 articles. Subsequently, 1 article was excluded due to the abstract, leaving 3 papers^27^, ^28^, ^29^ to be read in full, all being included for research (Fig. 1).

From the chosen descriptors, the databases were consulted and the results obtained are available in Table 3.

**Table 3:** Classification of references obtained from Pubmed, Scielo, Lilacs, Web of Science, Bireme and Scopus databases.

Descriptors	Nº	Excluded references	Reason	Selected	Database
(ototoxicity) AND (antineoplasic) AND (adults) AND (Childhood) AND (adolescent) AND (randomized controlled trial[pt] OR controlled clinical trial[pt] OR randomized controlled trials[mh] OR random allocation[mh] OR double-blind method[mh] OR singleblind method[mh] OR clinical trial[pt] OR clinical trials[mh] OR (“clinical trial”[tw]) OR ((singl*[tw] OR doubl*[tw] OR trebl*[tw] OR tripl*[tw]) AND (mask*[tw] OR blind*[tw])) OR (“latin square”[tw]) OR placebos[mh] OR placebo*[tw] OR random*[tw] OR research design[mh:noexp] OR follow-up studies[mh] OR prospective studies[mh] OR cross-over studies[mh] OR control*[tw] OR prospectiv*[tw] OR volunteer*[tw]) NOT (animal[mh] NOT human[mh])	7	4	Excluded by title (2); excluded by abstracts (1); excluded by repetition (1).	3	Pubmed
(ototoxicity) AND (antineoplasic) AND (adults) AND (Childhood) AND (adolescent) AND (randomized controlled trial[pt] OR controlled clinical trial[pt] OR randomized controlled trials[mh] OR random allocation[mh] OR double-blind method[mh] OR singleblind method[mh] OR clinical trial[pt] OR clinical trials[mh] OR (“clinical trial”[tw]) OR ((singl*[tw] OR doubl*[tw] OR trebl*[tw] OR tripl*[tw]) AND (mask*[tw] OR blind*[tw])) OR (“latin square”[tw]) OR placebos[mh] OR placebo*[tw] OR random*[tw] OR research design[mh:noexp] OR follow-up studies[mh] OR prospective studies[mh] OR cross-over studies[mh] OR control*[tw] OR prospectiv*[tw] OR volunteer*[tw]) NOT (animal[mh] NOT human[mh])	0	0	–	0	Lilacs
(ototoxicity) AND (antineoplasic) AND (adults) AND (Childhood) AND (adolescent) AND (randomized controlled trial[pt] OR controlled clinical trial[pt] OR randomized controlled trials[mh] OR random allocation[mh] OR double-blind method[mh] OR singleblind method[mh] OR clinical trial[pt] OR clinical trials[mh] OR (“clinical trial”[tw]) OR ((singl*[tw] OR doubl*[tw] OR trebl*[tw] OR tripl*[tw]) AND (mask*[tw] OR blind*[tw])) OR (“latin square”[tw]) OR placebos[mh] OR placebo*[tw] OR random*[tw] OR research design[mh:noexp] OR follow-up studies[mh] OR prospective studies[mh] OR cross-over studies[mh] OR control*[tw] OR prospectiv*[tw] OR volunteer*[tw]) NOT (animal[mh] NOT human[mh])	0	0	–	0	Scielo
(ototoxicity) AND (antineoplasic) AND (adults) AND (Childhood) AND (adolescent) AND (randomized controlled trial[pt] OR controlled clinical trial[pt] OR randomized controlled trials[mh] OR random allocation[mh] OR double-blind method[mh] OR singleblind method[mh] OR clinical trial[pt] OR clinical trials[mh] OR (“clinical trial”[tw]) OR ((singl*[tw] OR doubl*[tw] OR trebl*[tw] OR tripl*[tw]) AND (mask*[tw] OR blind*[tw])) OR (“latin square”[tw]) OR placebos[mh] OR placebo*[tw] OR random*[tw] OR research design[mh:noexp] OR follow-up studies[mh] OR prospective studies[mh] OR cross-over studies[mh] OR control*[tw] OR prospectiv*[tw] OR volunteer*[tw]) NOT (animal[mh] NOT human[mh])	0	0	–	0	WEB OF SCIENCE
(ototoxicity) AND (antineoplasic) AND (adults) AND (Childhood) AND (adolescent) AND (randomized controlled trial[pt] OR controlled clinical trial[pt] OR randomized controlled trials[mh] OR random allocation[mh] OR double-blind method[mh] OR singleblind method[mh] OR clinical trial[pt] OR clinical trials[mh] OR (“clinical trial”[tw]) OR ((singl*[tw] OR doubl*[tw] OR trebl*[tw] OR tripl*[tw]) AND (mask*[tw] OR blind*[tw])) OR (“latin square”[tw]) OR placebos[mh] OR placebo*[tw] OR random*[tw] OR research design[mh:noexp] OR follow-up Studies[mh] OR prospective studies[mh] OR cross-over studies[mh] OR control*[tw] OR prospectiv*[tw] OR volunteer*[tw]) NOT (animal[mh] NOT human[mh])	0	0	–	0	Bireme
(ototoxicity) AND (antineoplasic) AND (adults) AND (Childhood) AND (adolescent) AND (randomized controlled trial[pt] OR controlled clinical trial[pt] OR randomized controlled trials[mh] OR random allocation[mh] OR double-blind method[mh] OR singleblind method[mh] OR clinical trial[pt] OR clinical trials[mh] OR (“clinical trial”[tw]) OR ((singl*[tw] OR doubl*[tw] OR trebl*[tw] OR tripl*[tw]) AND (mask*[tw] OR blind*[tw])) OR (“latin square”[tw]) OR placebos[mh] OR placebo*[tw] OR random*[tw] OR research design[mh:noexp] OR follow-up studies[mh] OR prospective studies[mh] OR cross-over studies[mh] OR control*[tw] OR prospectiv*[tw] OR volunteer*[tw]) NOT (animal[mh] NOT human[mh])	0	0	–	0	SCOPUS
Total	7	4		3	Pubmed

Source: Developed by the authors.

The types of selected studies^27^, ^28^, ^29^ were cohort (2) and multicenter cohort (1). The analyzes were categorized according to the investigated theme, which indicated the intake of antineoplastic agents, the dosage used by the patients, as well as the ototoxic effects caused by the drugs. Regarding quality assessment, the studies obtained a score of 11 by Pithon et al.;^26^ therefore, they are of high quality for their inclusion and analysis. Table 4, Table 5 present the description of the research included in this systematic review, as well as the dosages of the drugs used in the oncological treatment of the subjects included in the three studies.

**Table 4:** Summary of selected articles.

Author/year/place of publication	Objective	n	Tests	Hearing loss classification	Results	Conclusion
Einarsson et al.^27^, 2010, Sweden	Investigate long-term hearing impairment in subjects who received platinum-based chemotherapy in childhood or adolescence.	15	PTA (0.125, 0.25, 0.5, 1, 2, 3, 4, 6 e 8 kHz)	Brock et al. (1991)	The results show that hearing loss, in subjects with ototoxicity, increased after the end of the treatment, also including the lower frequencies. The greatest deterioration in hearing thresholds, up to 55 dB HL, was found at frequencies above 2 kHz.	The conclusion of this study is that children and adolescents treated with platinum-based chemotherapy should undergo regular follow-up audiometric tests, also many years after the end of treatment. In addition, self-reported hearing impairment assessments should be made during and after chemotherapy
			Tympanometry Speech audiometry			
			Measurement of the handicap level (Hearing measurement scale — HMS)			
Clemens et al.^28^, 2016, Netherlands	To determine the frequency and determinants of ototoxicity in a large multicenter cohort of child cancer survivors who received platinum, but not cranial irradiation as treatment.	451	PTA (0.25, 0.5, 1, 2, 4 e 8 kHz)	Münster (Schmidt et al., 2007)	The general frequency of ototoxicity was 42%, observed in 45% of those treated with cisplatin, in 17% of those treated with carboplatin, and in 75% of those who received both drugs. The lowest age at the time of diagnosis, the highest total cumulative dose of cisplatin, and the concomitant treatment with furosemide were associated with ototoxicity.	Treatment with a higher total cumulative dose of cisplatin, younger age at diagnosis, and concomitant use of furosemide, independently, are associated with an increased risk of ototoxicity in subjects treated with platinum in childhood.
Clemens et al.^29^, 2017, Netherlands	To study the reversibility of ototoxicity after discontinuing treatment in a cohort of subjects treated with platinum with hearing loss at the end of cancer treatment.	168	PTA (0.25, 0.5, 1, 2, 4 e 8 kHz): age ≥5 years	Münster (Schmidt et al., 2007)	Of the 168 participants, 61 (36.3%) had hearing loss after completing chemotherapy, and none of these subjects showed improvement in hearing function until 28.8 years after discontinuing treatment.	Ototoxicity after platinum treatment may be irreversible and monitoring, as well as longitudinal clinical haring care, is required.
			Conditioned play audiometry: age between 2 and 5 years.			
			VRA in children aged 6 months to 2 years.			

Source: Einarsson et al.^27^ and Clemens et al.^28^, ^29^

PTA, Pure tone audiometry; VRA, Visual reinforcement audiometry.

**Table 5:** Cumulative doses of cisplatin and carboplatin and medications used concomitantly with chemotherapy.

Authors (year)	Cumulative dose	Cumulative dose	Cumulative dose	Concomitant medications
	Cisplatin (n)	Carboplatin (n)	Cisplatin + carboplatin (n)	
Einarsson et al.^27^ (2010)	NHG: varied from 180 to 690 mg/m^2^ (n = 5)	–	HLG: cisplatin = 320 mg/m^2^, carboplatin = 3000 mg/m^2^ (n = 1)	NCI
	HLG: ranged from 360 to 500 mg/m^2^ (n = 8)			
Clemens et al.^28^ (2016)	Ranged from 45 to 950 mg/m^2^ (n = 276)	Ranged from 104 to 9436 mg/m^2^ (n = 112)	Cisplatin = ranged from 80 to 570 mg/m^2^, carboplatin = ranged from 400 to 6043 mg/m^2^ (n = 63)	Vancomycin, gentamicin, tobramycin, furosemide (n = 285)
Clemens et al.^29^ (2017)^a^	Ranged from 180 to 900 mg/m^2^ (n = 46)	Ranged from 1288 to 3230 mg/m^2^ (n = 2)	Cisplatin = ranged from 300 to 570 mg/m^2^, carboplatin = ranged from 992 to 3938 mg/m^2^ (n = 13)	NI

Source: Einarsson et al.^27^ and Clemens et al.^28^, ^29^

N, sample number; NHG, normal hearing group; HLG, hearing loss group; NI, no information; mg/m^2^: milligram per square meter.

a Only subjects with hearing loss were included.

The sample size of the studies was quite heterogeneous, involving 15,^27^ 451,^28^ and 168^29^ evaluated cancer patients, all diagnosed in childhood, seen between 1985 and 2000, in the first and in the last two studies; cohort data (Long-term Effects after Childhood Cancer – LATER – group) between 1980 and 2012 were used (Table 4).

The age at diagnosis ranged from 0 to 18.9 years, and in the study by Einarsson et al.,^27^ the patients were divided into groups, and the average age was 15.7 years among subjects with normal hearing (n = 6) and 10.3 in the group with hearing loss (n = 9). In the other studies, the mean age at diagnosis was 4.9^28^ and 9.4 years.^29^ The mean time between the last dose of cisplatin and the post-treatment hearing assessment was clearly reported in only one of the studies, and ranged from 0.3 to 57.3 weeks, with an average time of 12.1 weeks in the group with hearing loss and 10.8 weeks among subjects with normal hearing.^27^

In all included studies, individuals were treated with cisplatin and carboplatin, alone or in combination, without cranial radiation. The cumulative doses of cisplatin, when given alone, varied from 45 to 950 mg/m^2^, those of carboplatin, also given alone, were between 104–9436 mg/m^2^ and when used in combination the cumulative doses of cisplatin varied from 80 to 570 mg/m^2^ and that of carboplatin from 400 to 6043 mg/m^2^ (Table 5).

In the study by Einarsson et al.,^27^ it was noticed that in the group with hearing impairment, 4 of the 6 individuals received a cumulative dose of cisplatin greater than 400 mg/m^2^, while in the group with normal hearing, only 3 of the 9 individuals received this same dose, however the subject who received the highest cumulative dose in the study (690 mg/m^2^) belonged to the group with normal hearing. When analyzing the cumulative doses of antineoplastic agents given to the 61 subjects with hearing loss in the most recent study,^29^ there was heterogeneity in the quantities, with the cumulative dose of cisplatin ranging from 180 to 900 mg/m^2^ and, when associated with carboplatin, it ranged from 300 to 570 mg/m^2^. The study by Clemens et al.^28^ found that the highest total cumulative dose of cisplatin (OR = 1.3; 95% CI: 1.2–1.5 per 100 mg/m^2^ increase) was associated with ototoxicity.

The consensus hearing assessment in the three studies was pure tone audiometry (PTA), although the frequency range showed significant differences, none of which aimed to assess high frequencies (Table 4). Regarding the criterion adopted for the analysis of hearing loss, one study used the Brock’s classification^30^ and the other two the Münster’s classification system.^31^

Regarding the time of audiological monitoring, 28 it was reported only in the last two studies, with an average of 5.9 (min.: 1.1 and max.: 27.2) years after the completion of treatment in one of the studies^29^ and a mean of 8.5 (min.: 0 and max.: 32.1) years in the other study.^28^ The general frequency of ototoxicity evidenced in the three studies ranged from 36.3%^29^ to 42%,^28^ being the first value referring to hearing alterations found after the end of treatment.The ototoxicity caused by cisplatin alone was 83.3%,^27^ 45%,^28^ and 75.4%,^29^ and that caused by carboplatin alone was 17%^28^ and 3.3%,^29^ while ototoxicity caused by the combined use both drugs was 16.6%,^27^ 75%,^28^ and 21.3%.^29^ One survey found that younger age at diagnosis, highest total cumulative dose of cisplatin, and combined treatment with furosemide are associated with an increased risk of ototoxicity.^28^ Hearing impairment was found after the end of chemotherapy treatment.

One of the studies^27^ demonstrated that hearing disorders occurred up to 22.3 years after the completion of treatment and involved high and low frequencies. In the other study,^29^ 36.3% of subjects treated for cancer had hearing loss on treatment discontinuation (Münster Grade 2b > 40 – ≤ 60 dB). In 39.3% (n = 24) of the subjects with hearing loss, there was an increase in Münster’s degree after an average time of 3.5 years (range: 1.1–21.3 years), with changes in hearing thresholds being observed even after an average time of 12.4 years (range 5.2–19.6 years) (n = 2), although the Münster’s score has remained unchanged over time, after an average time of 5.1 years (range 1.1–21.3 years) in 52.5% (n = 32) of subjects with hearing loss.

As for the PTA results, only the study using Brock’s classification^30^ described the average of some hearing thresholds and found, among the subjects with hearing loss, an average of pure tones for 3, 4, 6 and 8 kHz of the best ear of 66.9 dB and the worst ear of 74.8 dB HL. In contrast, among subjects with normal hearing, the average pure tone at the same frequencies was 1.9 dB HL in the best ear and 9.2 dB HL in the worst ear.^27^

## Discussion

This research aimed to present scientific evidence on the ototoxic effects of antineoplastic drugs, considering that the guiding question was based on verifying the effects and dose required for the ototoxicity of using antineoplastic drugs. It is worth mentioning that ototoxic drugs used in the treatment of cancer and the damage caused to the auditory system have aroused more and more interest to researchers, and the selection and implementation of appropriate procedures to monitor hearing may contribute to the adoption of appropriate measures regarding the ideal doses, the frequency of use, the combination or not of medications, minimizing the risks and damages, providing a better quality of life to these patients.

In all studies included in this research, the report of ototoxicity due to the use of antineoplastic agents in the treatment of patients was found, with the ototoxicity caused by cisplatin being the most evident, ranging from 45%^28^ to 83.3%,^27^ when used alone. These values are higher than those found in previous studies, in which the incidence of cisplatin-caused ototoxicity in children ranged from 22% to 70%.^32^, ^33^, ^34^, ^35^ A relatively recent publication investigating the hearing of 200 subjects treated for childhood cancer showed that treatment involving chemotherapy with cisplatin caused hearing loss in 41.9% of the right ears and 47.3% of the left ears, with an 11.7 times greater risk of hearing loss in the right ear and 17.6 times greater in the left ear, compared to patients not treated with cisplatin.^23^

In a study published in 2013, performed with a cohort of 112 children and 17 adults, of which 108 were treated with cisplatin, with 13 receiving carboplatin and 8 treated with both platinum compounds, showed that after therapy discontinuation, 47,3% of patients had hearing impairment, of which 55 children (49.1%) and 6 adults (42.1%).^15^ Cisplatin is widely used for chemotherapy, but is limited by cell resistance and possible serious side effects in tissues, which include nephrotoxicity, neurotoxicity, and ototoxicity. Ototoxicity occurs in the cochlear mechanosensory hair cells.^36^

Carboplatin ototoxicity was found in a study performed with 60 children with retinoblastoma who were treated with carboplatin associated with systemic vincristine. Twelve children (20%) developed ototoxicity at some point after the start of treatment, in 2 of them ototoxicity was reversible and in 10 (17%) it was irreversible.^14^ However, in another publication in which carboplatin was not used in combination with other ototoxic drugs, no hearing impairment was found.^37^ Carboplatin has an antitumor activity similar to cisplatin but with milder ototoxicity; however, it may be related to sensorineural hearing loss.^14^

In the studies included in the present study, individuals were treated with cisplatin and carboplatin, alone or in combination, however without cranial radiation, in order to rule out the participation of this treatment modality in hearing disorders. The effect of cisplatin on the inner ear is indisputable, and the combination of radiotherapy increases the likelihood of hearing loss.^38^, ^39^ The cumulative doses of cisplatin, when given alone, varied from 45 to 950 mg/m^2^, those of carboplatin also given alone were between 104–9436 mg/m^2^ and when used in combination the cumulative doses of cisplatin varied from 80 to 570 mg/m^2^ and that of carboplatin from 400 to 6043 mg/m^2^. Nitz et al.^15^ also showed higher cumulative doses of carboplatin when compared to cisplatin, both in subjects treated with the drugs alone or in combination. The average cumulative dose of carboplatin, when used alone, was 1500 mg/m^2^, the average cumulative dose of cisplatin was 360 mg/m^2^ and, when using the combined medications, the average cumulative doses of cisplatin and carboplatin were 240 mg/m^2^ and 1200 mg/m^2^, respectively.

Among the factors most highlighted as having the highest risk for developing platinum-induced hearing loss, the cumulative dosage of cisplatin greater than 400 mg/m^2^ stands out,^40^ although dosages above 200 mg/m² have already been shown to be ototoxic.^41^ Researchers argue that children treated with cisplatin at cumulative doses close to 400 mg/m^2^ need long-term surveillance to avoid neglecting hearing deficits. Carboplatin, at a standard dose, does not appear to be a significant risk factor for ototoxicity, even in patients who have already been treated with cisplatin.^32^

Cisplatin ototoxicity results in bilateral and symmetrical sensorineural hearing loss, worse at high frequencies (4–8 kHz),^16^, ^42^, ^43^ and tinnitus may also occur.^43^ In a study conducted with subjects cured of cancer, treated with cisplatin and combinations, the involvement occurred from 1 kHz onwards, with a marked increase from 6 kHz onwards.^21^ The degree of hearing loss is dose-dependent,^42^, ^44^ and is related to the frequency and method of assessment.^44^ The findings of the most recent study^29^ indicate that hearing loss caused by platinum-based antineoplastic agents is irreversible in the long term, since none of the subjects who developed hearing loss after treatment with antineoplastic agents showed progressive improvement in hearing function up to 28.8 years after stopping treatment. In another paper selected for this study,^27^ there was also no improvement in hearing loss in the evaluations performed during the follow-up.

One of the studies included in this systematic review showed that younger age at diagnosis is associated with an increased risk of ototoxicity,^28^ corroborating what was found in other studies,^14^, ^32^, ^33^ but contrasting with what was found in research that did not find age as a risk factor for ototoxicity.^15^ Li et al.^40^ found that children under the age of five at the time of treatment were 21 times more likely to acquire moderately severe high-frequency hearing loss compared to patients aged 15–20 years. Therefore, children treated with antineoplastic agents should routinely undergo long-term audiological monitoring.^14^

In the studies included in this review, the age at diagnosis ranged from 0 to 18.9 years, diverging between studies and also between findings in the literature, in which the average age at diagnosis was 13.56 (ranging from 10.26 to 16,27) years.^15^ The average time between the last chemotherapy and the hearing assessment was reported in one of the studies and ranged from 0.3 to 57.3 weeks, with an average time of 12.1 weeks in the group with hearing loss,^27^ less than that observed in another study that found an average interval between the end of the last chemotherapy course with cisplatin and carboplatin and the first post-treatment audiometry of 6.97 months (0.87–15.63 months).^15^

Another factor reported in scientific research suggests that genetics may be a relevant factor in ototoxicity, however the results are still contradictory and incipient.^45^ Screening for genetic predisposition to cisplatin ototoxicity can identify individuals at increased risk of hearing loss. Pharmacogenetic studies that investigate genetic variants have found mixed results, which may be related to the variability of patient populations and differentiated treatments.^46^

Regarding the criterion adopted for the analysis of hearing loss, one study^27^ used the Brock’s classification^30^ and the other two^28^, ^29^ used the Münster’s classification system.^31^ It is important to note that the different classifications and the difference in sensitivity between them can influence the calculated frequency. Brock’s classification^30^ has a favorable system for the classification of high frequency ototoxicity, while the Münster’s classification^31^ has better sensitivity and specificity.^28^ Clemens et al.^28^ cite that the frequency of ototoxicity evidenced was 42% using the Münster’s classification system,^31^ however when using Brock's classification criteria^30^ the frequency of ototoxicity suffered a significant variation, 29%.

The 2012 publication showed that different classification systems for ototoxicity showed good general agreement in identifying patients with this condition,^14^ however the classification systems used were: Brock et al.,^30^ National Cancer Institute Common Terminology Criteria for Adverse Events (NCI-CTCAE) version 3 (National Cancer Institute) and Children’s Cancer Group (CCG).^47^ Regarding hearing evaluation, it is noteworthy that all studies adopted conventional audiometry as a procedure and, although the frequency range has shown significant differences, none of them aimed to assess high frequencies. However, it is believed that high frequency audiometry should always be the procedure of choice, especially for monitoring patients exposed to ototoxic drugs, due to the initial involvement of high frequencies, characteristic of this pathology.

High frequency audiometry is considered an important tool for the detection and monitoring of hearing loss and assists in the detection of cisplatin ototoxicity, with frequencies of 12 kHz and 14 kHz being especially important.^48^ Studies carried out with patients treated with cisplatin and evaluated by means of conventional audiometry, as well as by means of high frequency audiometry, found auditory alteration mainly in frequencies from 6 to 9 kHz. 21, 49, 50 It is worth mentioning that the longer the medication remains in the body, the greater its harmful effect. However, individual differences regarding response to the toxic agent and variables that facilitate ototoxicity should be taken into account, such as family history of deafness, susceptibility to noise, among others.^21^

From all this analysis of scientific evidence, the importance of regular audiological monitoring during and after platinum-based treatment is highlighted, and it is also suggested to perform self-reported hearing impairment during and after chemotherapy.^27^

## Conclusion

The studies selected for this systematic review converged in their results, evidencing the effect of hearing changes after the use of platinum-based antineoplastic drugs, the ototoxicity caused by cisplatin being the most evident, and in many cases it can be irreversible.

Regarding the frequency of ototoxicity of antineoplastic drugs and the dose required to trigger these effects, an important heterogeneity was observed. The frequency of ototoxicity caused by cisplatin ranged from 45% to 83.3% and, when used in combination with carboplatin, ranged from 16.6% to 75%. In subjects with hearing loss the cumulative doses of cisplatin, when administered alone, ranged from 180 to 900 mg/m^2^, and when administered together with carboplatin it varied from 300 to 570 mg/m^2^, with the highest total cumulative dose of cisplatin being associated with ototoxicity.

## Conflicts of interest

The authors declare no conflicts of interest.
